# Demethoxycurcumin Is A Potent Inhibitor of P-Type ATPases from Diverse Kingdoms of Life

**DOI:** 10.1371/journal.pone.0163260

**Published:** 2016-09-19

**Authors:** Trong Tuan Dao, Pankaj Sehgal, Truong Thanh Tung, Jesper Vuust Møller, John Nielsen, Michael Palmgren, Søren Brøgger Christensen, Anja Thoe Fuglsang

**Affiliations:** 1 Department of Plant and Environmental Sciences, University of Copenhagen, Copenhagen, Denmark; 2 Department of Drug Design and Pharmacology, University of Copenhagen, Copenhagen, Denmark; 3 Department of Biomedicine, University of Aarhus, Aarhus, Denmark; Cinvestav-IPN, MEXICO

## Abstract

P-type ATPases catalyze the active transport of cations and phospholipids across biological membranes. Members of this large family are involved in a range of fundamental cellular processes. To date, a substantial number of P-type ATPase inhibitors have been characterized, some of which are used as drugs. In this work a library of natural compounds was screened and we first identified curcuminoids as plasma membrane H^+^-ATPases inhibitors in plant and fungal cells. We also found that some of the commercial curcumins contain several curcuminoids. Three of these were purified and, among the curcuminoids, demethoxycurcumin was the most potent inhibitor of all tested P-type ATPases from fungal (Pma1p; H^+^-ATPase), plant (AHA2; H^+^-ATPase) and animal (SERCA; Ca^2+^-ATPase) cells. All three curcuminoids acted as non-competitive antagonist to ATP and hence may bind to a highly conserved allosteric site of these pumps. Future research on biological effects of commercial preparations of curcumin should consider the heterogeneity of the material.

## Introduction

P-type ATPases constitute a large superfamily of enzymes that carry out pivotal processes in all kingdoms of life by pumping specific cations or phospholipids across lipid bilayers at the expense of one ATP molecule per cycle [1–5]. The characteristic structural features of the catalytic subunit of P-type ATPase are three cytosolic domains, comprising an actuator (A), a nucleotide binding (N), and a phosphorylation domain (P) that together with a multispan transmembrane domain, and in some cases additional regulatory cytosolic domains (R) at the N- or C-terminal ends [6–11] make up the pump functional unit. They are classified into five major families, P1 to P5-ATPases, which are further subdivided into subgroups based on their sequence motifs and transport specificity [1, 2, 12]. A hallmark of the P-type ATPases is the presence of an aspartic acid residue in a highly conserved motif DKTGT of the P-domain, which is phosphorylated by ATP during the reaction cycle [2–4, 13]. Some P-type ATPases are associated with transport of essential micronutrients such as Zn^2+^ and Cu^2+^(P1B-ATPases) and Ca^2+^ (P2A and P2B-ATPases). Cation transport by others generates an electrochemical gradient that in some cases can be used for secondary active transport e.g. plasma membrane (PM) H^+^-ATPases in plants and fungi (P3A) and Na^+^/K^+^-ATPases in animals (P2C), and for active transport of lipids (P4-ATPases) [2, 3, 14, 15]. Because of their critical role in the regulation of cellular metabolism, malfunctions of P-type ATPases are associated with a number of widespread diseases [16, 17].

Due to their important role in health and disease there is considerable interest in identification of drugs targeting P-type ATPases and, to date, a substantial number of P-type ATPase inhibitors have been synthesized or identified from natural sources [17]. Omeprazole is an example of a synthetic drug that is used to treat dyspeptic conditions and ouabain is a well known natural compound used to treat congestive heart failure [18, 19]. Mipsagargin, a prodrug of thapsigargin, a specific inhibitor of the sarco/encoplasmatic Ca^2+^-ATPase (SERCA), has in clinical trials showed promising effects against hepatocellular carcinoma [20]. Recently an allosteric activator of SERCA has been shown to lower fasting blood glucose, improve glucose tolerance and ameliorate hepatosteatosis in ob/ob mice [21].

Fungal P-type ATPases such as the PM H^+^-ATPase are considered as attractive targets for the development of new antifungal agents [22–24]. The potential of such agents might include preservation of food and conservation of crops. At the present, however, no promising lead compound has been found [17, 25–27].

Inhibition of plant PM H^+^-ATPase directly affects closure of the stomatal pores, which is used as a protective measure to help plants overcome extreme stress, such as drought, salinity or invasions of pathogens [28–32]. A well-described modulator of the PM H^+^-ATPase in the guard cells surrounding the stomatal pore through which plants breath is the fungal toxin fusicoccin [33, 34]. In conclusion, identification and characterization of inhibitors of P-type ATPases can be expected to be valuable for the discovery of drug leads and of new agents to protect plants and crops towards infections and for further studies on the structure and function of the P-type ATPase.

Turmeric rhizomes obtained from either *Curcuma longa* L. or *C*. *zanthorrhiza* Roxb. have been used in traditional Indian (Ayurveda), Arabian and Chinese medicine for millennia [35, 36]. The rhizomes are still included in the European Pharmacopoeia. The coloring and biological activities have been related to the presence of mainly curcuminoids with curcumin (CM, **1**), demethoxycurcumin (DMCM, **2**), bisdemethoxycurcumin (BDCM, **3**) as the main constituents and small amounts of other curcuminoids like cyclocurcumin [37, 38]. Even though some methods have been developed to separate these compounds on a preparative as well as an analytical scale, most commercially available samples consist of a mixture of curcuminoids [39–41]. Despite that the chemical community has been aware of the presence of several curcuminoids, the biological community has only to a limited extent paid attention to this fact when examining the biological activities of curcumin preparations.

Several publications assign the biological activity of turmeric rhizomes to CM, the main component of curcumin preparations. CM is believed to possess antibacterial and antifungal activity [42], to have antimalarial effects as well as serving in protection against other parasites [43, 44]. In a mouse model for Alzheimers disease CM decreases the level of oxidized proteins and interleukin 1-*β* [45]. A number of potential target molecules for CM have been identified such as growth factors, transcription factors, protein kinases, other enzymes (such as cyclooxygenase 2 and 5 lipoxygenase) [36] as well as P-type-ATPases [46–49]. Curcumin is undergoing phase I clinical trials as an adjuvant to improve the effectiveness of chemotherapeutic against breast cancer [50].

In our efforts to screen for small molecule inhibitors of P-type ATPases, and in particular PM H^+^-ATPases, we screened commercial curcumin preparations. An analysis of the product revealed that demethoxycurcumin (DMCM), one of the major curcuminoids in turmeric accounts for 15–20%, CM for about 60% and BDCM for about 5%. Many studies have used commercial curcumin preparations apparently without paying attention to the heterogeneity of the material. We have examined in detail the effects of the different curcuminoids CM, DMCM and BDCM. The compounds were tested on PM H^+^-ATPases from plant (AHA2), yeast (Pma1p) and the Ca^2+^-ATPase (SERCA), and they were all found to inhibit these P-type pumps as noncompetitive ATP antagonists. Furthermore, we were able to demonstrate antigrowth effects by DMCM on *Saccharomyces cerevisiae*. This effect is likely to be caused by the inhibition of the essential PM H^+^-ATPase.

## Materials and Methods

### Chemical Materials

Curcumin (cat #C7727), dibenzylideneacetone (cat#246425) and 6-shogaol (cat #39303) were purchased from Sigma-Aldrich.

### Yeast Strain and Plasmids

The *S*. *cerevisiae* strain RS-72 (*MATa ade1-100 his4-519 leu2-3*, *112*) [51] was transformed according to the lithium acetate/single-stranded carrier DNA/polyethylene glycol method [52], and cultured essentially as described previously [53]. In RS-72, the promoter of the endogenous yeast PM H^+^-ATPase *PMA1* gene was replaced by the galactose-dependent *GAL1* promoter. This strain grew in media containing galactose, whereas growth in glucose-based medium requires the complementation of the yeast PM H^+^-ATPase with a functional PM H^+^-ATPase expressed from a plasmid. A 2-micron yeast expression vector was used for expression of different versions of the *Arabidopsis thaliana* PM H^+^-ATPase *AHA2* (pMP 1625); a 92-amino acid C-terminal truncated mutation, *aha2∆92* (pMP 132); or the wild-type *S*. *cerevisiae* PM H^+^-ATPase *PMA1* (pMP 400) placed under the control of the *PMA1* promoter [54, 55]. The use of RS-72 allows for purification of microsomes with a very low endogenous ATPase activity compared to RS-72 transformed with an H^+^-ATPase carrying plasmid [53, 56].

### Purification of Plasma Membrane H^+^-ATPases

Yeast expressing AHA2 and Pma1p PM H^+^-ATPase, respectively, were grown and harvested essentially as described [57]. The cells were grown with glucose as carbon source in order to ensure than no endogenous plasma membrane H^+^-ATPase is expressed. Microsomes and plasma membranes were isolated [57] with all manipulations performed at 4°C. The final pellets containing the isolated membranes were collected and homogenized in GTED20 buffer (20% [v/v] glycerol, 10 mM Tris-HCl pH 7.5, 1 mM EDTA pH 8.0, 1 mM DTT) and stored at—80°C.

### Purification of Spinach Plasma Membrane

Baby spinach (*Spinacia oleracea*) leaves (50 g) were cut into small pieces and homogenized. Subsequently plasma membranes were isolated by two-phase partitioning according to [58]. Plasma membranes were collected and homogenized in the phase buffer containing 330 mM sucrose, 5 mM potassium phosphate pH 7.8, 2 mM KCl, 0.1 mM EDTA, and 1 mM DTT and stored at—80°C.

### Procedures for Experiments with SERCA

Sarcoplasmic reticulum vesicles, prepared from the leg and back muscles of rabbits, were used to obtain purified SERCA1a by extraction with a low concentration of deoxycholate according to established methods [59]. The resulting membrane fragments were used for enzymatic assays to test the effect of the curcuminoid compounds on ATP hydrolysis spectrophotometrically by NADH oxidation with an ATP regenerating system [60].

### Screening of Chemical Library

A number of 160 compounds were chosen from an in-house library of natural products. The compounds represent a diversity of chemical structures and physicochemical properties important for drugs, such as molecular weight < 500, clogP < 5 and tPSA < 100 [61]. All the tested compounds are listed in S1 Fig. Stock solutions of these compounds (10 mM) were prepared in 50% of DMSO. The compounds were tested up to 100 μM final concentration. Compounds that inhibited PM H^+^-ATPase activity more than 50 percent of the control were selected and retested at lower concentrations.

### Isolation of Curcumin Analogs

**Fig 1:**
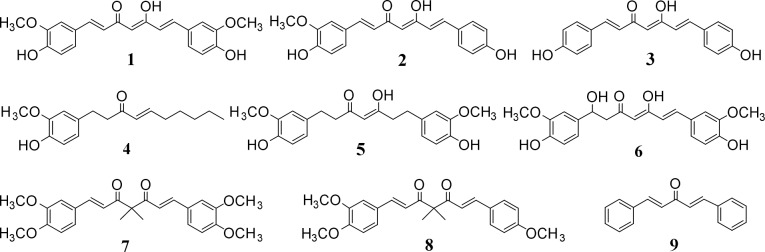
Curcumin analogs employed in this study. Curcumin (Sigma-Aldrich) was initially identified as a hit in an *in vitro* screen for inhibitors of the plasma membrane H^+^-ATPase. Curcumin (CM, **1**) demethoxycurcumin (DMCM, **2**), bisdemethoxycurcumin (BDCM, **3**) and 1,5-dihydroxy-1,7-bis(4-hydroxy-3-methoxyphenyl)-4,6-heptadien-3-one (**6**) were then purified as previous described [39, 62]. 6-shogaol (**4**) and dibenzylideneacetone (**9**) were obtained from Sigma-Aldrich. Tetrahydrocurcumin (**5**), 1,7-bis(3',4'-dimethoxyphenyl)-4,4-dimethyl-1,6-heptadien-3,5-dione (**7**) and 1-(3',4'-dimethoxyphenyl)-4,4-dimethyl-7-(4'-methoxyphenyl)-1,6-heptadien-3,5-dione (**8**) were synthesized as previously described in the literature[63].

Curcumin (CM, **1**), demethoxycurcumin (DMCM, **2**), bisdemethoxycurcumin (BDCM, **3**) and 1,5-dihydroxy-1,7-bis(4-hydroxy-3-methoxyphenyl)-4,6-heptadien-3-one (**6**) were purified as previous described [39, 62] (Fig 1). Tetrahydrocurcumin (**5**), 1,7-bis(3',4'-dimethoxyphenyl)-4,4-dimethyl-1,6-heptadien-3,5-dione (**7**) and 1-(3',4'-dimethoxyphenyl)-4,4-dimethyl-7-(4'-methoxyphenyl)-1,6-heptadien-3,5-dione (**8**) were synthesized as described by Takeuchi et al. [63] (Fig 1). The compounds were dissolved in DMSO and stored as small aliquots at—20°C.

### PM H^+^-ATPase Assays

ATPase activity was determined by the Baginski assay as described previously [64] using 2–5 μg of PM protein. The assays were performed at 30°C in 300 μL volumes with 3 mM ATP, pH 6.5, for determination of AHA2 activity, or with 5 mM ATP, pH 5.9 for Pma1p activity. The assay medium 20 mM MOPS, 50 mM KNO_3_ (to inhibit vacuolar ATPase), 5 mM NaN_3_ (to inhibit mitochondrial ATPase), 3.5 mM Na_2_MoO_4_ (to inhibit acid phosphatase), 1 mM Mg^2+^ free in solution, and the indicated concentrations of MgATP. IC_50_ values for compound inhibition were determined by pre incubating PM with different concentrations of compound for 30 min at room temperature. For kinetics studies, the concentration of ATP was varied between 0.125 and 8 mM using an ATP regenerating system (5 mM phosphoenolpyruvate and 50 μg/mL pyruvate kinase).

### Proton Pumping Assay

Inside-out vesicles were created from spinach plasma membranes by adding the detergent Brij-58 to the buffer. Proton transport into the vesicles was determined as described [58, 64] by monitoring fluorescence quenching (excitation at 412 nm, emission at 480 nm) of 9-amino-6-chloro-2-methoxyacridine (ACMA). The reaction medium contained 20 mM MOPS-KOH, pH 7.0, 40 mM K_2_SO_4_, 25 mM KNO_3_, 2 mM ATP, 1 μM ACMA, 60 nM valinomycin and 0.05% (w/v) Brij-58. Proton pumping reactions were started by the addition of MgSO_4_ to a final concentration of 2 mM, and the proton gradient was collapsed by the addition of 10 μM nigericin.

### Drop Tests

Transformed yeast cells, cultured on synthetic galactose (SG) agar medium supplemented with adenine 40 μg/mL and L-histidine 20 μg/mL (SGAH plates) for 3 days at 30°C, were suspended in H_2_O to 1x solution (OD_660_ = 1). Serial dilutions of 0.1, 0.01, 0.001 x of the cells were then prepared and spotted adjacently onto synthetic minimal media plates containing either 2% galactose or 2% glucose at pH 5.5 (SGAH or SDAH), incorporated with different concentrations of tested compound. Growth results were recorded after incubation for 3 days at 30°C. Minimum inhibitory concentrations (MIC values) were determined as the lowest concentrations of compounds where no visible growth of cells was observed.

### Protein Determination

Protein concentrations were determined by the method of Bradford employing bovine serum albumin as a reference [65].

### Kinetic and Dose-Response Analysis

The kinetic of inhibition for DMCM was determined by monitoring the rate of ATP hydrolysis reaction in the presence of increasing ATP substrate concentration. All kinetic data were gathered from experiments performed in the cuvette-based assay, as described above. The data were used in Hanes-Woolf analysis followed by fitting to the appropriate Michaelis-Menten inhibition equation (competitive, noncompetitive, uncompetitive, or mix type inhibition). The kinetic parameters *V*_max_, *K*_m_, *K*_i_ were estimated by nonlinear regression using GraphPad Prism 5.0 (GraphPad Software Inc.). For evaluation of inhibitor potency, IC_50_ values were calculated by using the equation:

$$A=\frac{100}{1+10^{(Log{IC}_{50}-LogC)\times H}}$$

where *A* is the observed % ATPase activity in the presence of compound; *C*, the compound concentration; IC_50_, the concentration that caused 50% inhibition of the maximal compound effect; and *H*, the slope factor.

### Statistics

All experiments were performed at least three independent times, each with three replicates. Values are presented as mean ± S.E.M. unless otherwise stated. P-values were calculated one- or two-way analysis of variance (ANOVA) followed by Bonferroni post-hoc testing using GraphPad Prism 5.0. P < 0.05 was considered statistically significant. *, p < 0.05; **, p < 0.01; ***, p < 0.001.

## Results

### Screening of a Chemical Library for Inhibitors of PM H^+^-ATPases

A library of 160 natural products was screened for the ability to inhibit fungal (Pma1p) and plant (AHA2) PM H^+^-ATPase. The selected library encompassed a diversity of structures possessing a broad range of physicochemical properties important for drug action. From the initial data, curcumin (Sigma-Aldrich, cat #C7727) was identified as the best hit, showing more than 95% of the inhibition at 100 μM as compared to the control (S1 Fig). An analysis of the commercially available curcumin revealed that the product consisted of approximately 80% of CM, 15% of DMCM and a number of minor curcuminoids including BDCM as demonstrated by HPLC (S2 Fig). This encouraged us to purify various curcuminoids (CM, DMCM and BDCM) present in the commercial curcumin for establishing the range of potency for each constituent.

### Effects of Curcumin Analogs on the PM H^+^-ATPases Activity

**Fig 2:**
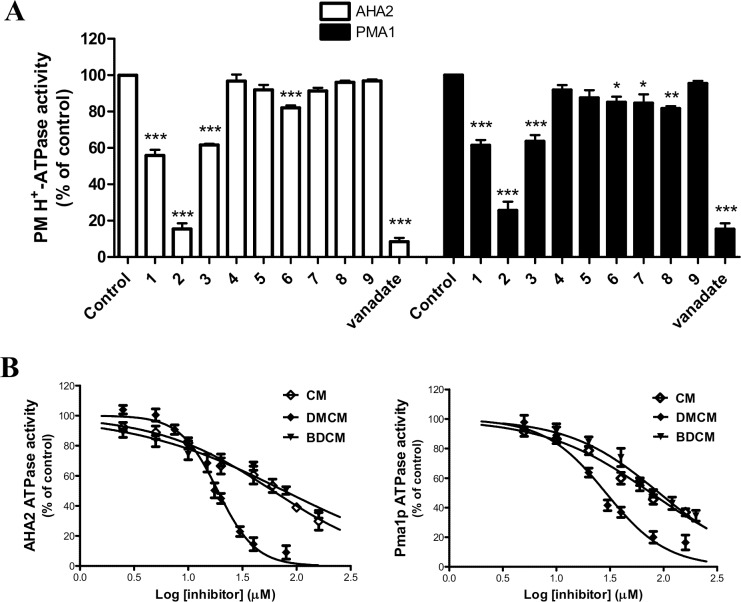
Curcumin analogs inhibit plasma membrane H^+^-ATPases activity in a dose dependent manner. (**A**) ATPase activity measured on yeast plasma membranes from yeast strains expressing either *A*. *thaliana* PM H^+^-ATPase isoform AHA2 (left panel) or the endogenous yeast plasma membrane Pma1p (right panel) in the presence of 50 μM of the curcumin analogs, compounds **1**–**9,** or vanadate, a P-type ATPase inhibitor. Values are mean ± S.E. (*n* = 3). Student’s *t* tests:*, p < 0.05; **, p < 0.01; ***, p < 0.001 relative to the control. (**B**) Dose-dependent inhibition of H^+^-ATPase activity of AHA2 (left) and Pma1p (right). Experiments were carried out in triplicates at different concentrations of CM (**1**), DMCM (**2**) and BDCM (**3**). Data were analyzed by using nonlinear regression tool and fitted to log(inhibitor) vs. normalized response (variable slope) for determination of IC_50_ values.

A series of nine curcumin analogs (Fig 1), which have different substitution patterns on the aryl moiety (**1**, **2**, **3**), different numbers of conjugated double bonds (**1**, **5**, **6**), phenol groups (**1**, **4**), or the central keto-enol functionalities (**1**, **4**, **9**) or blocked enolization (**1**, **7**, **8**) [66] were evaluated for their potential to inhibit PM H^+^-ATPases. The inhibitory effects of each compound on PM H^+^-ATPases activity were determined experimentally at a final concentration of 50 μM on recombinant plant (AHA2) or fungal (Pma1p) PMH^+^-ATPase enzyme, respectively. Only three of the curcumin analogs: CM, DMCM and BDCM significantly inhibited these enzymes (Fig 2A), among which DMCM (**2**) exhibited the most pronounced effect, while CM and BDCM were about half as effective. Interestingly these compounds were equally effective inhibitors of both plant and yeast PM H^+^-ATPase enzymes, whereas the other tested analogs did not exhibit any effect on AHA2 and Pma1p activity (Fig 2A). These preliminary structure activity observations suggest that the central keto-enol moiety and surrounding conjugated double bonds are chemical features important for the inhibitory activity towards the PM H^+^-ATPases.

### Inhibitory Effects on PM H^+^-ATPases by DMCM

To further evaluate the specific inhibitory activity of the three analogs on PM H^+^-ATPase activity, concentration response curves were generated (Fig 2B). The activity of AHA2 was reduced by DMCM in a concentration-dependent manner with IC_50_ of 18.7 μM, which is a more potent effect than that of CM and BDMC (IC_50_, 60.4 and 76.7 μM, respectively) (Table 1). The same pattern of inhibitory potencies was observed for Pma1p with IC_50_ values of 28.9, 76.1 and 90.6 μM, respectively (Fig 2B and Table 1). Interestingly, the structure of DMCM containing a methoxy moiety at only one of the two phenyl rings significantly enhanced the inhibitory activity (IC_50_ value) by a 2- to 4-fold lower value, in comparison to CM which has two *p*-hydroxy-*m*-methoxyphenyl rings or BDCM with only *p*-hydroxyphenyl rings. The inhibition curves were fitted with a variable slope factor H. The H factor for DMCM inhibition of AHA2 and Pma1p was 2.4 and 1.5 respectively, in contrast to the numbers for CM and BDCM that were close to 1. This suggests a cooperative binding of DMCM.

**Table 1:** IC_50_ values for compounds 1–3 in ATP hydrolysis assays.

Compound	AHA2	Pma1p	SERCA
CM (**1**)	60.4 ± 1.1	76.1 ± 1.0	3.9 ± 0.4
DMCM (**2**)	18.7 ± 1.0	28.9 ± 1.0	2.6 ± 0.9
BDCM (**3**)	76.7± 1.1	90.6 ± 1.1	9.4 ± 1.6

All values are presented as the mean of three independent experiments ± S.E., and indicated in μM.

### Effects of Curcuminoids on Proton Transport in Plasma Membrane Vesicles from Spinach Leaves

**Fig 3:**
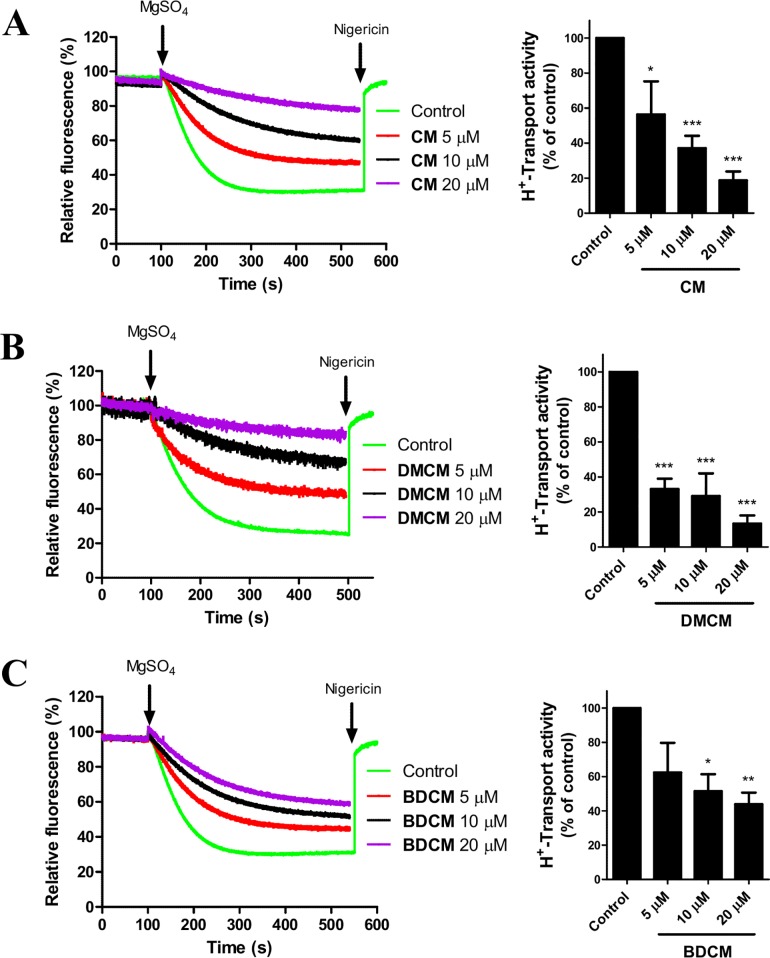
Curcumin analogs also inhibit proton pumping. (**A**-**C**) Proton mediated accumulation of ACMA in spinach plasma membrane vesicles after incubating with various concentrations of CM (**1**), DMCM (**2**) and BDCM (**3**). ATP stimulated H^+^-pumping is initiated upon addition of MgSO_4_. One representative of three independent experiments is shown for each compound (left panels). Proton transport activity was determined as initial rates of fluorescence quenching of ACMA probe (right panels). Values are mean ± S.E. (*n* = 3). Student’s *t* tests:*, p < 0.05; **, p < 0.01; ***, p < 0.001 relative to the control.

PM H^+^-ATPases utilize the energy released from ATP phosphorylation followed by hydrolysis to pump protons across the plasma membrane generating an electrochemical gradient required for nutrient and water uptakes by plant and fungi [28, 34]. Because of the tight coupling between ATP hydrolysis and proton transport [67, 68], we next studied the effects of curcuminoids on H^+^ pumping activity by using PM vesicles isolated from spinach leaves. Fig 3 shows the quenched fluorescence caused by proton accumulation inside the vesicles in response to treatment with the three curcuminoids. Again DMCM exhibited the strongest effect compared to CM and BDCM. The H^+^ pumping activity was dramatically reversed in the concentration range 5–20 μM of DMCM. The order of efficiency among the three compounds (Fig 3) was similar to the above ATP hydrolysis results and therefore strongly confirms the inhibition effects on PM H^+^-ATPases of these compounds.

### Mechanism of DMCM Inhibition

**Fig 4:**
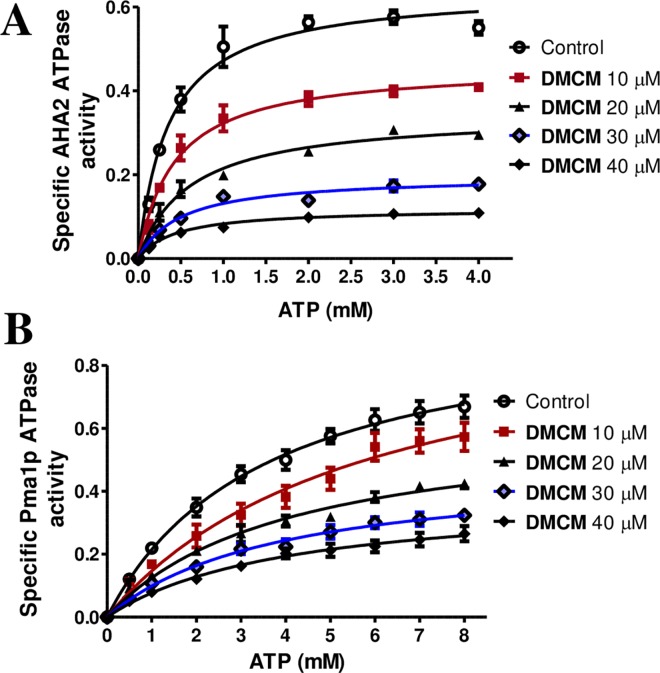
Kinetic analysis of PM H^+^-ATPases inhibition by DMCM. ATP hydrolysis rate was plotted as function of [ATP] in the presence of the indicated amounts of DMCM; Yeast expressing *A*. *thaliana* PM H^+^-ATPase isoform AHA2 (**A**) and the endogenous yeast isoform Pma1p (**B**). Error bars represent standard errors of the mean for three independent trials.

To characterize the mode of ATPase inhibition by DMCM, we performed enzyme kinetics with increasing concentrations of the substrate ATP (0.125–8 mM) in the presence of various concentrations of DMCM (0, 10, 20, 30 and 40 μM). Total ATP hydrolysis activity of AHA2 and Pma1p is depicted in Fig 4. An initial Hanes-Woolf plot indicated a non-competitive inhibition (not shown) the curves were therefore fitted by the appropriate nonlinear regression (with noncompetitive equation) using GraphPad Prism. We found that the apparent *K*_m_ values for ATP remained at a constant level, independent of the addition of DMCM. This was observed for both AHA2 and Pma1p with *K*_m_ values of 0.4 and 4.1 μM, respectively. The *V*_max_ values, on the other hand, were decreased due to increasing concentrations of DMCM (Fig 4 and Table 2). We estimated the *K*_i_ to be 14.7 and 27.7 μM for AHA2 and Pma1p respectively (Table 3), corresponding to the previously estimated IC_50_ values. These kinetic data suggest that DMCM behaves as a noncompetitive inhibitor of ATP.

**Table 2:** Effect of DMCM on the ATP hydrolysis of the PM H^+^-ATPases.

Demethoxycurcumin (μM)	AHA2		Pma1p	
	*K*_m_ (mM)	*V*_max_ (μM/mg/min)	*K*_m_ (mM)	*V*_max_ (μM/mg/min)
0	0.36 ± 0.05	0.64 ± 0.02	3.55 ± 0.48	0.98 ± 0.06
10	0.43 ± 0.06	0.46 ± 0.02	5.85 ± 1.45	1.00 ± 0.13
20	0.60 ± 0.08	0.35 ± 0.01	4.06 ± 0.65	0.63 ± 0.05
30	0.49 ± 0.08	0.19 ± 0.01	3.85 ± 0.71	0.48 ± 0.04
40	0.44 ± 0.09	0.11 ± 0.01	4.09 ± 0.89	0.39 ± 0.04

The ATP hydrolytic activity was measured with various concentrations of ATP and the kinetic constants (*V*_max_, *K*_m_ values) were determined from nonlinear regression of the Michaelis-Menten equation. Values are mean ± S.E. (*n* = 3).

**Table 3:** Effects of compounds 1–3 on PM H^+^-ATPases *in vitro* and in *in vivo* drop test assay.

Compound	In Vitro		Drop Test	
	AHA2 (*K*_i_, μM)	Pma1p (*K*_i_, μM)	AHA2 (MIC, μM)	Pma1p (MIC, μM)
CM (**1**)	ND	ND	40–50	60–70
DMCM (**2**)	14.7 ± 0.7	27.7 ± 1.2	20–30	40–50
BDCM (**3**)	ND	ND	200–210	210–220

The inhibition constants (*K*_i_ values) were estimated by fitting data to a nonlinear regression analysis using noncompetitive model. The values are mean ± S.E. (*n* = 3) and ND indicates not detected. Minimum inhibitory concentrations (MIC values) were determined by the lowest concentrations of CM (**1**), DMCM (**2**) and BDCM (**3**) where no visible growth of cells was observed.

### Effects of DMCM on SERCA Activity

**Fig 5:**
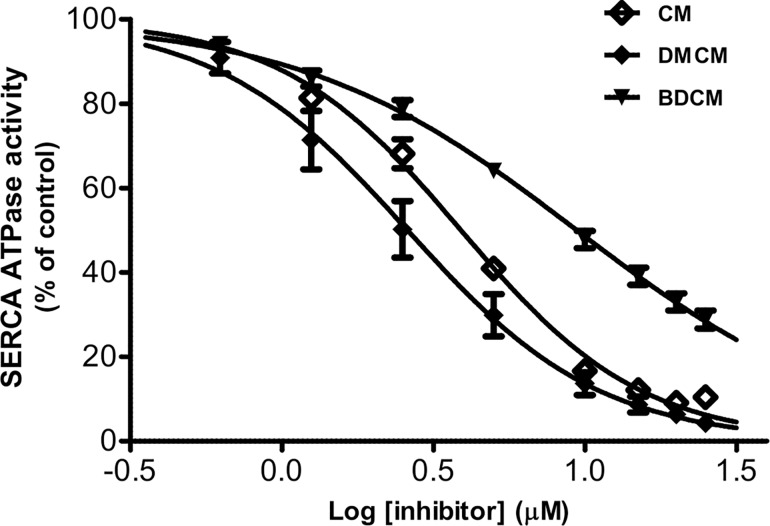
Dose-dependent inhibition of SERCA Ca^2+^-ATPase activity. Inhibition of ATP hydrolytic activity by addition of increasing amounts of compounds. Experiments were carried out in triplicate at different concentrations of CM (**1**), DMCM (**2**) and BDCM (**3**). Data were analyzed by using nonlinear regression tool and fitted to log(inhibitor) vs. normalized response (variable slope) for determination of IC_50_ values.

AHA2 and Pma1p are members of the P-type ATPase superfamily to which SERCA also belongs. Previously the effect of commercial curcumin on SERCA has been broadly investigated [46–48]. In none of these studies, however, has the homogeneity of the purchased CM been verified. Analysis of CM batches bought from different vendors revealed that many products consisted of CM-DMCM-BDCM in the approximate ratio of 80:15:5 (S2 Fig). All of this prompted us to analyze the inhibitory effect of the three curcumin analogs found in curcumin purchased from Sigma on SERCA activity. As the result of this test we could show that DMCM treatment had the most potent inhibitory effect on the ATPase activity of the purified and leaky preparations of SERCA (which we used to avoid complicating effects of intravesicular accumulation of Ca^2+^). From these experiments we extract an IC_50_ value of 2.6 μM from the dose dependent response curve in comparison to CM and BDCM (IC_50_, 3.9 and 9.4 μM, respectively) (Fig 5 and Table 1). Thus the inhibitory effects of these compounds on SERCA were found to be stronger, but with retention of the same order of potencies as observed on AHA2 and Pma1p pumps. These data, in conjunction with the report of an inhibitory effect of curcumin on Na^+^/K^+^-ATPase [49] suggest that DMCM may modulate different members of P-type ATPases *in vitro* by a common mechanism binding to a highly conserved region [69].

### Effects of Curcuminoids on Yeast Growth

**Fig 6:**
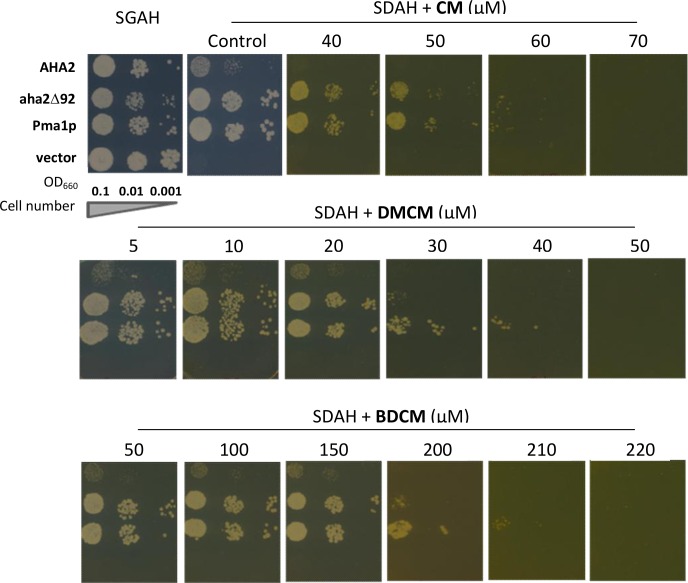
Drop tests showing the sensitive response of *S*. *cerevisiae* mutant cells (encoding AHA2, aha2∆92 or Pma1p) to compounds 1–3. Serial dilutions of the yeast cultures are spotted on galactose (YPG) or glucose (YPD) media containing the indicated concentrations of CM (**1**), DMCM (**2**) or BDCM (**3**). Growth results were recorded after incubation for 3 days at 30°C. Results shown are representative data of three independent experiments for each compound.

As PM H^+^-ATPases are essential for the growth of fungi, we used the yeast strain RS-72 as a model to test the potency of the curcuminoids. In *S*. *cerevisiae* strain RS-72, expression of the endogenous PM H^+^-ATPase PMA1 is dependent on the addition of galactose to the media enabling the possibility of expressing and testing other PM H^+^-ATPase homologues. RS-72 transformed with an empty vector (as control) is not able to grow in glucose containing media (Fig 6). RS-72 expressing either *Arabidopsis* homologues (wild-type: *AHA2* and a truncated mutation: *aha2∆92*) or wild-type *PMA1* from *S*. *cerevisiae* were grown on media containing increasing concentrations of the three compounds in question. The enzyme aha2∆92 is an activated form of AHA2 due to the removal of an autoinhibitory domain. Yeast cells dependent on heterologously expressed aha2∆92 grow better than cells expressing wild-type AHA2, which grow poorly compared to yeast expressing wild-type Pma1p. All the tested strains showed sensitivity to the added compounds (Fig 6). Again DMCM was the most effective inhibitor compared to CM and BDCM. Minimum inhibitory concentrations (MIC values) were approximately 30, 50 and 200 μM for DMCM, CM and BDCM, respectively (Fig 6 and Table 3) demonstrating a convincing positive correlation between the enzyme assay and the growth experiments.

## Discussion

### PM H^+^-ATPase Inhibition and the Antifungal Effect of Curcuminoids

Curcuminoids originate from rhizomes of *C*. *longa*. The dried and grounded rhizomes are commonly used as spices in food and in health products. Many studies have reported that the curcuminoids possess antifungal activity [42], and also have synergistic properties with other well known agents [70, 71]. However, very few studies have compared the activities of the three major curcuminoids. The present study provides insight into the mode of action of three curcuminoids and demonstrates an inhibitory effect on Pma1p, correlated with reduced fungal growth. This result is fully consistent with a recent report defining the pH differences in *Candida* culture medium in presence and absence of CM, which suggested curcumin to regulate H^+^- extrusion by PM ATPases [72].

In addition, we show that curcuminoids also inhibit plant PM H^+^-ATPase. This effect could be of interest in order to protect plants during pathogenic infections. In plants, several pathogenic bacteria enter the leaf through the stomatal pore and closure of the stomatal pore by inhibition of the PM H^+^-ATPase is one of the defense mechanisms exploited by plants upon recognition of pathogenic bacteria [29–32]. Also pretreatment of plants with curcumin revealed fungicidal activity towards selected plant pathogenic fungi [73], if this effect was due to a direct effect on the fungi or the ability to infect via the stomatal pore is not known. The low toxicity of the curcuminoids towards humans makes them interesting agents for developments of antifungal agents. The higher activity of DMCM makes this the most interesting lead compound to be used in organic agriculture and food preservation.

### DMCM Is a Potent SERCA Pump Inhibitor

Inhibitory effects of CM and some of its analogs, including BDCM, on SERCA have been broadly investigated. In the present study we compared the effect of DMCM with CM and BDCM on the basis of Ca^2+^-ATPase activity per se of the SERCA pump.

Interestingly, DMCM exhibited the strongest effect on SERCA among the tested curcuminoids with an IC_50_ value of 2.6 μM compared to IC_50_ values of 3.9 and 9.4 μM for CM and BDCM, respectively. Numerous studies to date have shown the pharmacological safety and efficacy of CM, however, it exhibits poor bioavailability due to fast metabolism primarily through reduction followed by conjugation to glucuronic or sulfuric acid [74]. Although the structural difference between CM and DMCM is a minor one (namely the presence or absence of a methoxy group), the chemical characteristics of DMCM are more stable [75]. Our data might inspire to future investigations of the performance of DMCM in prostate and colorectal cancers related to SERCA activity [76, 77].

### Structure Activity Relationships of Curcuminoids on ATPase Activity

In the current study, we have investigated the structure activity relationship of curcumin analogs and discovered molecular characteristics important for the effects against ATPase. The study has included the role of the hydroxyl aryl moiety, conjugated double bonds and the central keto-enol moiety. Although a previous study on SERCA has emphasized the importance of phenolic -OH at the 4 position and the non-essential role of *o*-methoxy group on the aryl ring [46], our data showed the more complicated interaction with this methoxy group. DMCM with only one methoxy group significantly enhanced the inhibitory activity, whereas CM and BDCM possessing two or no methoxy groups, respectively, were less active. This finding might be related to the asymmetrical nature of DMCM in contrast to CM and BDCM. In DMCM the two oxygen atoms at the aliphatic chain (O-3 and O-5) are not chemically identical and form a predominant tautomer B2 [78]. The *o*-methoxy group influences the electron density on the central keto-enol group, which in turn might affect its binding ability to ATPase proteins.

### Curcuminoids Are General P-Type ATPase Inhibitors

Inhibitors of P-type ATPases are important drugs [17]. Of particular importance are digoxin, ouabain [18, 19, 79], thapsigargin [80, 81] and cyclopiazonic acid [82], which are all derived from natural products. Turmeric, a spice with multiple pharmacological utilities [83] has been shown to possess a mild inhibitory effect on Na^+^/K^+^-ATPase [49], mammalianCa^2+^-ATPase [46–48] and a malaria parasite orthologue PfATP6 [84]. In this study we have reported DMCM as a potent inhibitor of SERCA (P2 type) and PM H^+^-ATPase (P3 type). These results and previous work on both SERCA [46–48] and Na^+^/K^+^-ATPase [49] lead us to consider DMCM and other curcuminoids as general P-type ATPase inhibitors that also modulate other members of the P-type ATPase superfamily. Our kinetic study revealed that DMCM is a non-competitive antagonist to ATP and hence may bind to a highly conserved allosteric site of these pumps. This is accordance with the previous findings for CM binding on SERCA [46–48]. Additionally our data suggested that DMCM might bind in a cooperative manner or that two binding sites are present. A similar observation is made in [48] where the authors suggest that binding of curcumin to the Ca^2+^-ATPase induces a conformational change, which then blocks the ATP binding.

In conclusion, our study reveals that curcuminoids target P-type ATPases present in fungi, plants and animals, and have antifungal properties. In spite of the inhibition of P-type ATPases they are not toxic for mammals [85], which can be explained by fast metabolism into glucuronides and sulfates [74, 86]. The structure activity relationship analysis of curcumin analogs against these ATPases provides crucial information for drug design and discovery process. Among the active curcuminoids present in the commercial preparation of curcumin, DMCM is the most potent inhibitor of all tested P-type ATPases. Future studies on biological effects of curcuminoids thus should consider the heterogeneity of the commercial samples of the compounds.

## Supporting Information

S1 Appendix ^1^H NMR and ^13^C NMR spectra for CM in acetone-*d*_*6*_. (TIF)

S2 Appendix ^1^H NMR and ^13^C NMR spectra for DMCM in acetone-*d*_*6*_. (TIF)

S3 Appendix ^1^H NMR and ^13^C NMR spectra for BDCM in acetone-*d*_*6*_. (TIF)

S1 Fig Screening of 163 compounds for their ability to inhibit plasma membrane H^+^-ATPase activity. ATPase activity measured in the presence of 100 μM of the indicated compounds. The effect is presented relative to an untreated sample. A sample containing the well-described inhibitor of P-type ATPases, vanadate, is included as control. Below the diagram all tested compounds are listed.
(TIFF)

S2 Fig HPLC analysis of commercially available curcumin. Analytical HPLC profiles of compounds **1**–**3** (**A**-**C**), and curcumins Sigma #C7727 (**D**), Abcam #120618 (**E**) and Aldrich #238384 (**F**) were performed using a Waters system: DAD detector 390 nm, column RP C18 (150 x 4.6 mm), flow rate 0.8 mL/min, injection volumn 5 μL of 0.1 mg/min sample solution, solvent gradient 44.9% MeCN, 55% H_2_O and 0.1% formic acid to 98.9% MeCN, 1% H_2_O and 0.1% formic acid over 15 min.
(TIFF)

## References

[1] Bublitz M, Morth JP, Nissen P. P-type ATPases at a glance (vol 124, pg 2515, 2011). J Cell Sci. 2011;124(22):3917–. 10.1242/jcs.102921 . 21768325

[2] Axelsen KB, Palmgren MG. Evolution of substrate specificities in the P-type ATPase superfamily. J Mol Evol. 1998;46(1):84–101. 9419228 10.1007/pl00006286

[3] Kuhlbrandt W. Biology, structure and mechanism of P-type ATPases. Nat Rev Mol Cell Bio. 2004;5(4):282–95. 10.1038/nrm1354 . 15071553

[4] Møller JV, Juul B, le Maire M. Structural organization, ion transport, and energy transduction of P- type ATPases. Biochim Biophys Acta. 1996;1286(1):1–51. 8634322 10.1016/0304-4157(95)00017-8

[5] Apell HJ. How do P-type ATPases transport ions? Bioelectrochemistry. 2004;63(1–2):149–56. 10.1016/j.bioelechem.2003.09.021 . 15110265

[6] Toyoshima C, Nakasako M, Nomura H, Ogawa H. Crystal structure of the calcium pump of sarcoplasmic reticulum at 2.6 angstrom resolution. Nature. 2000;405(6787):647–55. 10.1038/35015017 . 10864315

[7] Palmgren MG, Sommarin M, Serrano R, Larsson C. Identification of an autoinhibitory domain in the C-terminal region of the plant plasma membrane H^+^-ATPase. J Biol Chem. 1991;266(30):20470–5. 1834646

[8] Portillo F, de Larrinoa IF, Serrano R. Deletion analysis of yeast plasma membrane H^+^-ATPase and identification of a regulatory domain at the carboxyl-terminus. Febs Lett. 1989;247(2):381–5. . 2523820 10.1016/0014-5793(89)81375-4

[9] Rasi-Caldogno F, Carnelli A, De Michelis MI. Identification of the Plasma Membrane Ca^2+^-ATPase and of Its Autoinhibitory Domain. Plant Physiol. 1995;108(1):105–13. 12228456 10.1104/pp.108.1.105 PMC157310

[10] Hwang I, Harper JF, Liang F, Sze H. Calmodulin activation of an endoplasmic reticulum-located calcium pump involves an interaction with the N-terminal autoinhibitory domain. Plant Physiol. 2000;122(1):157–68. 10631259 10.1104/pp.122.1.157 PMC58854

[11] Olesen C, Picard M, Winther A-ML, Gyrup C, Morth JP, Oxvig C, et al The structural basis of calcium transport by the calcium pump. Nature. 2007;450(7172):1036–42. 18075584 10.1038/nature06418

[12] Bublitz M, Poulsen H, Morth JP, Nissen P. In and out of the cation pumps: P-Type ATPase structure revisited. Curr Opin Struc Biol. 2010;20(4):431–9. 10.1016/j.sbi.2010.06.007 . 20634056

[13] Lutsenko S, Kaplan JH. Organization of P-Type Atpases—Significance of Structural Diversity. Biochemistry-Us. 1995;34(48):15607–13. 10.1021/Bi00048a001 . 7495787

[14] Pedersen CN, Axelsen KB, Harper JF, Palmgren MG. Evolution of plant P-type ATPases. Frontiers in plant science. 2012;3:31 10.3389/fpls.2012.00031 22629273 PMC3355532

[15] Palmgren M, Baekgaard L, Lopez-Marques R, Fuglsang A. Plasma Membrane ATPases 2011. 177–92 p.

[16] Brini M, Cali T, Ottolini D, Carafoli E. The plasma membrane calcium pump in health and disease. The FEBS journal. 2013;280(21):5385–97. 10.1111/febs.12193 . 23413890

[17] Yatime L, Buch-Pedersen MJ, Musgaard M, Morth JP, Winther AML, Pedersen BP, et al P-type ATPases as drug targets: Tools for medicine and science. Bba-Bioenergetics. 2009;1787(4):207–20. 10.1016/j.bbabio.2008.12.019 . 19388138

[18] Ogawa H, Shinoda T, Cornelius F, Toyoshima C. Crystal structure of the sodium-potassium pump (Na^+^, K^+^-ATPase) with bound potassium and ouabain. Proceedings of the National Academy of Sciences. 2009;106(33):13742–7. 10.1073/pnas.0907054106 PMC2728964 19666591

[19] Yatime L, Laursen M, Morth JP, Esmann M, Nissen P, Fedosova NU. Structural insights into the high affinity binding of cardiotonic steroids to the Na^+^, K^+^-ATPase. J Struct Biol. 2011;174(2):296–306. 10.1016/j.jsb.2010.12.004 21182963

[20] Doan NTQ, Christensen SB. Thapsigargin, Origin, Chemistry, Structure-Activity Relationships and Prodrug Development. Curr Pharm Design. 2015;21(38):5501–17. 10.2174/1381612821666151002112824 . 26429715

[21] Kang S, Dahl R, Hsieh W, Shin AC, Zsebo KM, Buettner C, et al Small Molecular Allosteric Activator of the Sarco/Endoplasmic Reticulum Ca^2+^-ATPase (SERCA) Attenuates Diabetes and Metabolic Disorders. J Biol Chem. 2015 Epub 2015/12/25. 10.1074/jbc.M115.705012 . 26702054 PMC4777852

[22] Monk BC, Perlin DS. Fungal plasma membrane proton pumps as promising new antifungal targets. Critical reviews in microbiology. 1994;20(3):209–23. 7802957 10.3109/10408419409114555

[23] Seto-Young D, Monk B, Mason AB, Perlin DS. Exploring an antifungal target in the plasma membrane H^+^-ATPase of fungi. Biochimica et Biophysica Acta (BBA)-Biomembranes. 1997;1326(2):249–56. 9218555 10.1016/s0005-2736(97)00028-x

[24] Soteropoulos P, Vaz T, Santangelo R, Paderu P, Huang DY, Tamás MJ, et al Molecular Characterization of the Plasma Membrane H^+^-ATPase, an Antifungal Target inCryptococcus neoformans. Antimicrob Agents Chemother. 2000;44(9):2349–55. 10952578 10.1128/aac.44.9.2349-2355.2000 PMC90068

[25] Billack B, Piętka-Ottlik M, Santoro M, Nicholson S, Młochowski J, Lau-Cam C. Evaluation of the antifungal and plasma membrane H^+^-ATPase inhibitory action of ebselen and two ebselen analogs in S. cerevisiae cultures. J Enzyme Inhib Med Chem. 2010;25(3):312–7. 10.3109/14756360903179419 20210698

[26] Kongstad KT, Wubshet SG, Kjellerup L, Winther A-ML, Stærk D. Fungal plasma membrane H^+^-ATPase inhibitory activity of o-hydroxybenzylated flavanones and chalcones from Uvaria chamae P. Beauv. Fitoterapia. 2015;105:102–6. 10.1016/j.fitote.2015.06.013 26102180

[27] Kongstad KT, Wubshet SG, Johannesen A, Kjellerup L, Winther A-ML, ger AK, et al High-Resolution Screening Combined with HPLC-HRMS-SPE-NMR for Identification of Fungal Plasma Membrane H^+^-ATPase Inhibitors from Plants. J Agric Food Chem. 2014;62(24):5595–602. 10.1021/jf501605z 24830509

[28] Palmgren MG. Plant plasma membrane H⁺-ATPases: Powerhouses for nutrient uptake. Annual Review of Plant Physiology and Plant Molecular Biology. 2001;52:817–45. . 10.1146/annurev.arplant.52.1.817 11337417

[29] Melotto M, Underwood W, Koczan J, Nomura K, He S. Plant stomata function in innate immunity against bacterial invasion. Cell. 2006;126(5):969–80. 16959575 10.1016/j.cell.2006.06.054

[30] Underwood W, Melotto M, He SY. Role of plant stomata in bacterial invasion. Cell Microbiol. 2007;9(7):1621–9. 17419713 10.1111/j.1462-5822.2007.00938.x

[31] Melotto M, Underwood W, He SY. Role of stomata in plant innate immunity and foliar bacterial diseases. Annual review of phytopathology. 2008;46:101 10.1146/annurev.phyto.121107.104959 18422426 PMC2613263

[32] Liu J, Elmore JM, Fuglsang AT, Palmgren MG, Staskawicz BJ, Coaker G. RIN4 functions with plasma membrane H⁺-ATPases to regulate stomatal apertures during pathogen attack. Plos Biol. 2009;7(6):e1000139 Epub 2009/07/01. 10.1371/journal.pbio.1000139 19564897 PMC2694982

[33] Baunsgaard L, Fuglsang AT, Jahn T, Korthout HAAJ, de Boer AH, Palmgren MG. The 14-3-3 proteins associate with the plant plasma membrane H^+^-ATPase to generate a fusicoccin binding complex and a fusicoccin responsive system. Plant Journal. 1998;13(5):661–71. 10.1046/J.1365-313x.1998.00083.X . 9681008

[34] Falhof J, Pedersen JT, Fuglsang AT, Palmgren M. Plasma membrane H-ATPase regulation in the center of plant physiology. Mol Plant. 2015 10.1016/j.molp.2015.11.002 . 26584714

[35] Huang KC. The pharmacology of Chinese herbs: CRC press; 1998.

[36] Shishodia S, Sethi G, Aggarwal BB. Curcumin: getting back to the roots. Ann N Y Acad Sci. 2005;1056(1):206–17. 16387689 10.1196/annals.1352.010

[37] Kiuchi F, Goto Y, Sugimoto N, Akao N, Kondo K, Tsuda Y. Nematocidal activity of turmeric: synergistic action of curcuminoids. Chem Pharm Bull (Tokyo). 1993;41(9):1640–3. Epub 1993/09/01. . 8221978 10.1248/cpb.41.1640

[38] Priyadarsini KI. The chemistry of curcumin: from extraction to therapeutic agent. Molecules. 2014;19(12):20091–112. Epub 2014/12/04. 10.3390/molecules191220091 . 25470276 PMC6270789

[39] Rasmussen HB, Christensen SB, Kvist LP, Karazmi A. A simple and efficient separation of the curcumins, the antiprotozoal constituents of Curcuma longa. Planta Med. 2000;66(4):396–8. Epub 2000/06/24. 10.1055/s-2000-8533 . 10865470

[40] Chauhan S, Singh B, Agrawala S. Estimation of curcuminoids in Curcuma longa by HPLC and spectrophotometric methods. Indian J Pharm Sci. 1999;61(1):58.

[41] Ashraf K, Mujeeb M, Ahmad A, Ahmad N, Amir M. Determination of Curcuminoids in Curcuma longa Linn. by UPLC/Q-TOF-MS: An Application in Turmeric Cultivation. J Chromatogr Sci. 2015. 10.1093/chromsci/bmv023 25838167

[42] Moghadamtousi SZ, Kadir HA, Hassandarvish P, Tajik H, Abubakar S, Zandi K. A review on antibacterial, antiviral, and antifungal activity of curcumin. BioMed research international. 2014;2014. 10.1155/2014/186864 PMC4022204 24877064

[43] Reddy RC, Vatsala PG, Keshamouni VG, Padmanaban G, Rangarajan PN. Curcumin for malaria therapy. Biochem Biophys Res Commun. 2005;326(2):472–4. 15582601 10.1016/j.bbrc.2004.11.051

[44] Fournet A, Muñoz V. Natural products as trypanocidal, antileishmanial and antimalarial drugs. Curr Top Med Chem. 2002;2(11):1215–37. 12171582 10.2174/1568026023393011

[45] Lim GP, Chu T, Yang F, Beech W, Frautschy SA, Cole GM. The curry spice curcumin reduces oxidative damage and amyloid pathology in an Alzheimer transgenic mouse. The Journal of Neuroscience. 2001;21(21):8370–7. 11606625 10.1523/JNEUROSCI.21-21-08370.2001 PMC6762797

[46] Logan-Smith MJ, Lockyer PJ, East JM, Lee AG. Curcumin, a molecule that inhibits the Ca^2+^-ATPase of sarcoplasmic reticulum but increases the rate of accumulation of Ca^2+^. J Biol Chem. 2001;276(50):46905–11. 10.1074/jbc.M108778200 . 11592968

[47] Sumbilla C, Lewis D, Hammerschmidt T, Inesi G. The slippage of the Ca^2+^ pump and its control by anions and curcumin in skeletal and cardiac sarcoplasmic reticulum. J Biol Chem. 2002;277(16):13900–6. 10.1074/jbc.M111155200 . 11844792

[48] Bilmen JG, Khan SZ, Javed M-U-H, Michelangeli F. Inhibition of the SERCA Ca2+ pumps by curcumin: curcumin putatively stabilizes the interaction between the nucleotide-binding and phosphorylation domains in the absence of ATP. Eur J Biochem. 2001;268(23):6318–27. 10.1046/j.0014-2956.2001.02589.x . 11733029

[49] Mahmmoud YA. Curcumin modulation of Na,K-ATPase: phosphoenzyme accumulation, decreased K^+^ occlusion, and inhibition of hydrolytic activity. British journal of pharmacology. 2005;145(2):236–45. 10.1038/sj.bjp.0706185 15753945 PMC1576134

[50] Bayet-Robert M, Kwiatkowski F, Leheurteur M, Gachon F, Planchat E, Abrial C, et al Phase I dose escalation trial of docetaxel plus curcumin in patients with advanced and metastatic breast cancer. Cancer biology & therapy. 2010;9(1):8–14. . 19901561 10.4161/cbt.9.1.10392

[51] Cid A, Perona R, Serrano R. Replacement of the promoter of the yeast plasma membrane ATPase gene by a galactose-dependent promoter and its physiological consequences. Curr Genet. 1987;12(2):105–10. 2966684 10.1007/BF00434664

[52] Gietz RD, Woods RA. Transformation of yeast by lithium acetate/single-stranded carrier DNA/polyethylene glycol method. Methods Enzymol. 2002;350:87–96. Epub 2002/06/21. . 12073338 10.1016/s0076-6879(02)50957-5

[53] Regenberg B, Villalba JM, Lanfermeijer FC, Palmgren MG. C-terminal deletion analysis of plant plasma membrane H^+^-ATPase: yeast as a model system for solute transport across the plant plasma membrane. Plant Cell. 1995;7(10):1655–66. 7580256 10.1105/tpc.7.10.1655 PMC161027

[54] Rudashevskaya EL, Ye J, Jensen ON, Fuglsang AT, Palmgren MG. Phosphosite Mapping of P-type Plasma Membrane H^+^-ATPase in Homologous and Heterologous Environments. J Biol Chem. 2012;287(7):4904–13. 10.1074/jbc.M111.307264 . 22174420 PMC3281613

[55] Palmgren MG, Christensen G. Complementation in situ of the yeast plasma membrane H^+^-ATPase gene *pma1* by an H^+^-ATPase gene from a heterologous species. Febs Lett. 1993;317(3):216–22. 8425607 10.1016/0014-5793(93)81279-9

[56] Villalba JM, Palmgren MG, Berberian GE, Ferguson C, Serrano R. Functional expression of plant plasma membrane H^+^-ATPase in yeast endoplasmic reticulum. J Biol Chem. 1992;267(17):12341–9. 1534807

[57] Axelsen KB, Venema K, Jahn T, Baunsgaard L, Palmgren MG. Molecular dissection of the C-terminal regulatory domain of the plant plasma membrane H^+^-ATPase AHA2: mapping of residues that when altered give rise to an activated enzyme. Biochemistry-Us. 1999;38(22):7227–34. 10.1021/bi982482l 10353834

[58] Lund A, Fuglsang AT. Purification of plant plasma membranes by two-phase partitioning and measurement of H^+^ pumping. Methods Mol Biol. 2012;913:217–23. 10.1007/978-1-61779-986-0_14 . 22895762

[59] Moller JV, Olesen C. Preparation of Ca^2+^-ATPase1a Enzyme from Rabbit Sarcoplasmic Reticulum. Methods Mol Biol. 2016;1377:11–7. 10.1007/978-1-4939-3179-8_3 . 26695018

[60] Sehgal P, Olesen C, Moller JV. ATPase Activity Measurements by an Enzyme-Coupled Spectrophotometric Assay. Methods Mol Biol. 2016;1377:105–9. 10.1007/978-1-4939-3179-8_11 . 26695026

[61] Lipinski CA, Lombardo F, Dominy BW, Feeney PJ. Experimental and computational approaches to estimate solubility and permeability in drug discovery and development settings. Advanced drug delivery reviews. 2001;46(1–3):3–26. Epub 2001/03/22. . 11259830 10.1016/s0169-409x(00)00129-0

[62] Dao TT, Nguyen PH, Won HK, Kim EH, Park J, Won BY, et al Curcuminoids from Curcuma longa and their inhibitory activities on influenza A neuraminidases. Food Chem. 2012;134(1):21–8.

[63] Takeuchi T, Ishidoh T, Iijima H, Kuriyama I, Shimazaki N, Koiwai O, et al Structural relationship of curcumin derivatives binding to the BRCT domain of human DNA polymerase λ. Genes Cells. 2006;11(3):223–35. 16483311 10.1111/j.1365-2443.2006.00937.x

[64] Venema K, Palmgren MG. Metabolic modulation of transport coupling ratio in yeast plasma membrane H^+^-ATPase. J Biol Chem. 1995;270(33):19659–67. 7642655 10.1074/jbc.270.33.19659

[65] Bradford MM. A rapid and sensitive method for the quantitation of microgram quantities of protein utilizing the principle of protein-dye binding. Anal Biochem. 1976;72:248–54. Epub 1976/05/07. S0003269776699996 [pii]. . 942051 10.1016/0003-2697(76)90527-3

[66] Padhye S, Chavan D, Pandey S, Deshpande J, Swamy KV, Sarkar FH. Perspectives on chemopreventive and therapeutic potential of curcumin analogs in medicinal chemistry. Mini Rev Med Chem. 2010;10(5):372–87. Epub 2010/04/08. ; PubMed Central PMCID: PMCPmc3084451. 20370702 10.2174/138955710791330891 PMC3084451

[67] Briskin DP. Intermediate reaction states of the red beet plasma membrane ATPase. Arch Biochem Biophys. 1986;248(1):106–15. 2942108 10.1016/0003-9861(86)90406-6

[68] Buch-Pedersen MJ, Rudashevskaya EL, Berner TS, Venema K, Palmgren MG. Potassium as an intrinsic uncoupler of the plasma membrane H^+^-ATPase. J Biol Chem. 2006;281(50):38285–92. 10.1074/Jbc.M604781200 . 17056603

[69] Sorensen TL, Clausen JD, Jensen AM, Vilsen B, Moller JV, Andersen JP, et al Localization of a K^+^ -binding site involved in dephosphorylation of the sarcoplasmic reticulum Ca^2+^ -ATPase. J Biol Chem. 2004;279(45):46355–8. 10.1074/jbc.C400414200 . 15383548

[70] Sharma M, Manoharlal R, Negi AS, Prasad R. Synergistic anticandidal activity of pure polyphenol curcumin I in combination with azoles and polyenes generates reactive oxygen species leading to apoptosis. FEMS yeast research. 2010;10(5):570–8. 10.1111/j.1567-1364.2010.00637.x 20528949

[71] Tsao S-M, Yin M-C. Enhanced inhibitory effect from interaction of curcumin with amphotericin B or fluconazole against Candida species. Journal of Food and Drug Analysis. 2000;8(3).

[72] Neelofar K, Shreaz S, Rimple B, Muralidhar S, Nikhat M, Khan LA. Curcumin as a promising anticandidal of clinical interest. Can J Microbiol. 2011;57(3):204–10. 10.1139/W10-117 21358761

[73] Kim M-k, Choi G-j, Lee H-s. Fungicidal property of Curcuma longa L. rhizome-derived curcumin against phytopathogenic fungi in a greenhouse. J Agric Food Chem. 2003;51(6):1578–81. 12617587 10.1021/jf0210369

[74] Anand P, Kunnumakkara AB, Newman RA, Aggarwal BB. Bioavailability of curcumin: problems and promises. Molecular pharmaceutics. 2007;4(6):807–18. 10.1021/mp700113r . 17999464

[75] Pfeiffer E, Hohle S, Solyom AM, Metzler M. Studies on the stability of turmeric constituents. J Food Eng. 2003;56(2–3):257–9. Pii S0260-8774(02)00264-9 10.1016/S0260-8774(02)00264-9 .

[76] Fan L, Li A, Li W, Cai P, Yang B, Zhang M, et al Novel role of Sarco/endoplasmic reticulum calcium ATPase 2 in development of colorectal cancer and its regulation by F36, a curcumin analog. Biomed Pharmacother. 2014;68(8):1141–8. 10.1016/j.biopha.2014.10.014 25458791

[77] Denmeade SR, Isaacs JT. The SERCA pump as a therapeutic target: making a “smart bomb” for prostate cancer. Cancer Biol Ther. 2005;4(1):21–9. 10.4161/cbt.4.1.1505 15662118

[78] Ostrowski W, Śniecikowska L, Hoffmann M, Frański R. Demethoxycurcumin-Metal Complexes: Fragmentation and Comparison with Curcumin-Metal Complexes, as Studied by ESI-MS/MS. Journal of Spectroscopy. 2013;2013.

[79] Schultheis PJ, Wallick E, Lingrel J. Kinetic analysis of ouabain binding to native and mutated forms of Na, K-ATPase and identification of a new region involved in cardiac glycoside interactions. J Biol Chem. 1993;268(30):22686–94. 8226778

[80] Denmeade SR, Jakobsen CM, Janssen S, Khan SR, Garrett ES, Lilja H, et al Prostate-specific antigen-activated thapsigargin prodrug as targeted therapy for prostate cancer. J Natl Cancer Inst. 2003;95(13):990–1000. 12837835 10.1093/jnci/95.13.990

[81] Sagara Y, Inesi G. Inhibition of the sarcoplasmic reticulum Ca^2+^ transport ATPase by thapsigargin at subnanomolar concentrations. J Biol Chem. 1991;266(21):13503–6. 1830305

[82] Laursen M, Bublitz M, Moncoq K, Olesen C, Møller JV, Young HS, et al Cyclopiazonic acid is complexed to a divalent metal ion when bound to the sarcoplasmic reticulum Ca^2+^-ATPase. J Biol Chem. 2009;284(20):13513–8. 10.1074/jbc.C900031200 19289472 PMC2679452

[83] Anand P, Thomas SG, Kunnumakkara AB, Sundaram C, Harikumar KB, Sung B, et al Biological activities of curcumin and its analogues (Congeners) made by man and Mother Nature. Biochemical pharmacology. 2008;76(11):1590–611. 10.1016/j.bcp.2008.08.008 . 18775680

[84] Shukla A, Singh A, Singh A, Pathak L, Shrivastava N, Tripathi P, et al Inhibition of P. falciparum pfATP6 by curcumin and its derivatives: A bioinformatic Study. Cell Mol Biol. 2012;58(1):182–6. 23273210

[85] Ganiger S, Malleshappa HN, Krishnappa H, Rajashekhar G, Ramakrishna Rao V, Sullivan F. A two generation reproductive toxicity study with curcumin, turmeric yellow, in Wistar rats. Food and chemical toxicology: an international journal published for the British Industrial Biological Research Association. 2007;45(1):64–9. 10.1016/j.fct.2006.07.016 . 16987575

[86] Vareed SK, Kakarala M, Ruffin MT, Crowell JA, Normolle DP, Djuric Z, et al Pharmacokinetics of curcumin conjugate metabolites in healthy human subjects. Cancer epidemiology, biomarkers & prevention: a publication of the American Association for Cancer Research, cosponsored by the American Society of Preventive Oncology. 2008;17(6):1411–7. 10.1158/1055-9965.EPI-07-2693 18559556 PMC4138955

