# Supplementary figures and images for: Demethoxycurcumin Is A Potent Inhibitor of P-Type ATPases from Diverse Kingdoms of Life
Source: PLoS One. 2016 Sep 19;11(9):e0163260. doi: 10.1371/journal.pone.0163260 (PMC5028038; doi:10.1371/journal.pone.0163260)

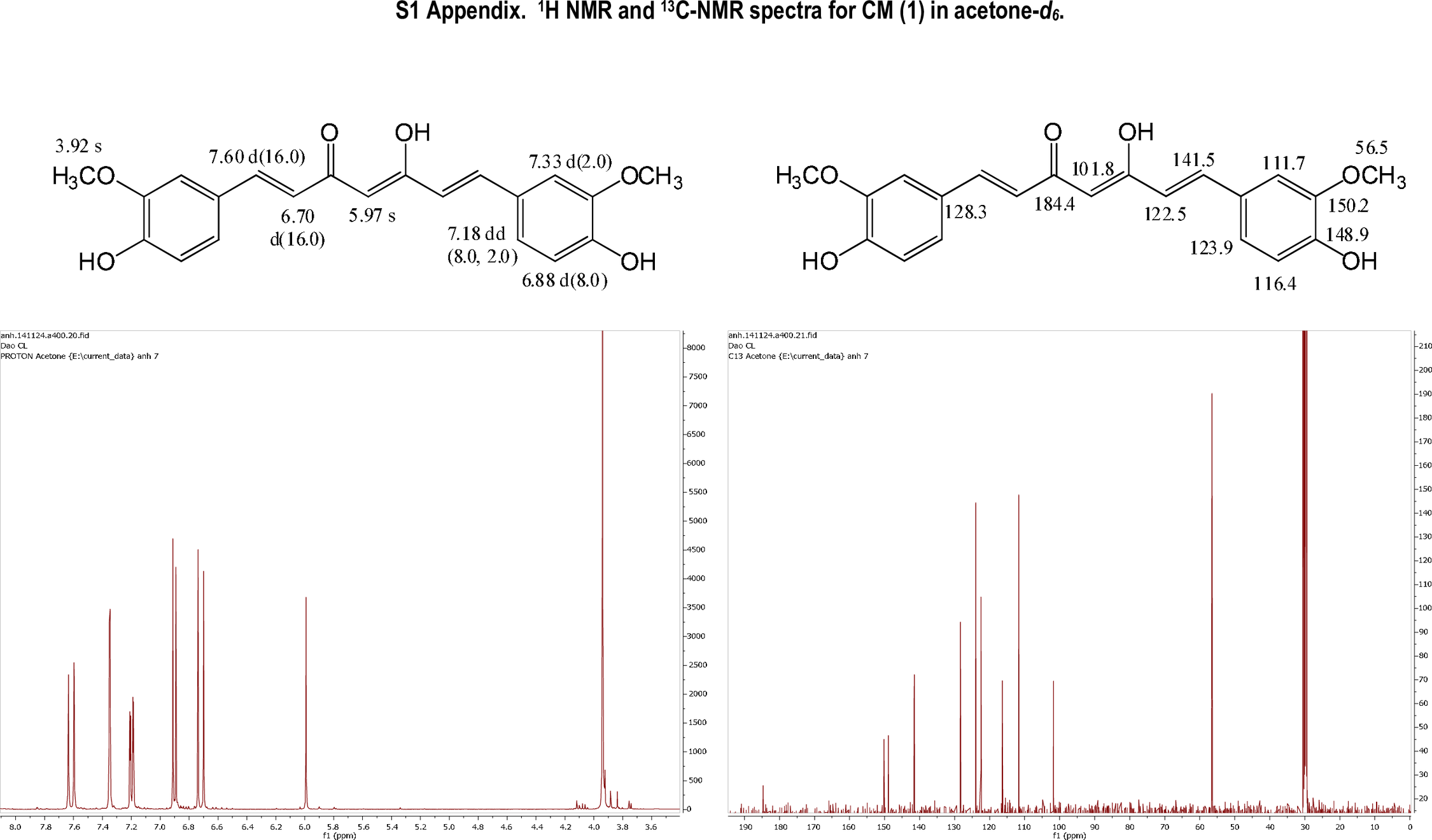

Supplement: S1 Appendix — (TIF) [file pone.0163260.s001.tif]

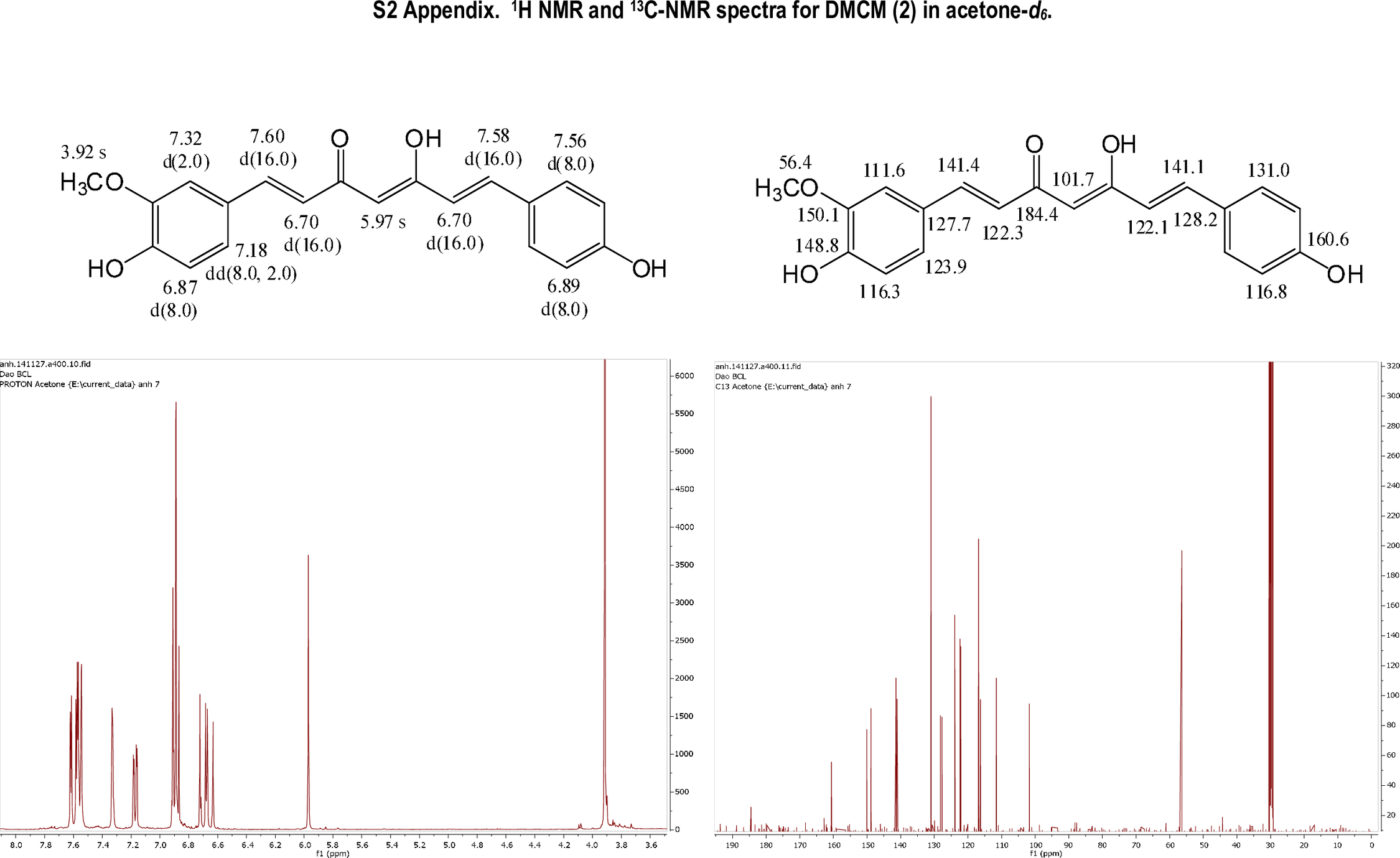

Supplement: S2 Appendix — (TIF) [file pone.0163260.s002.tif]

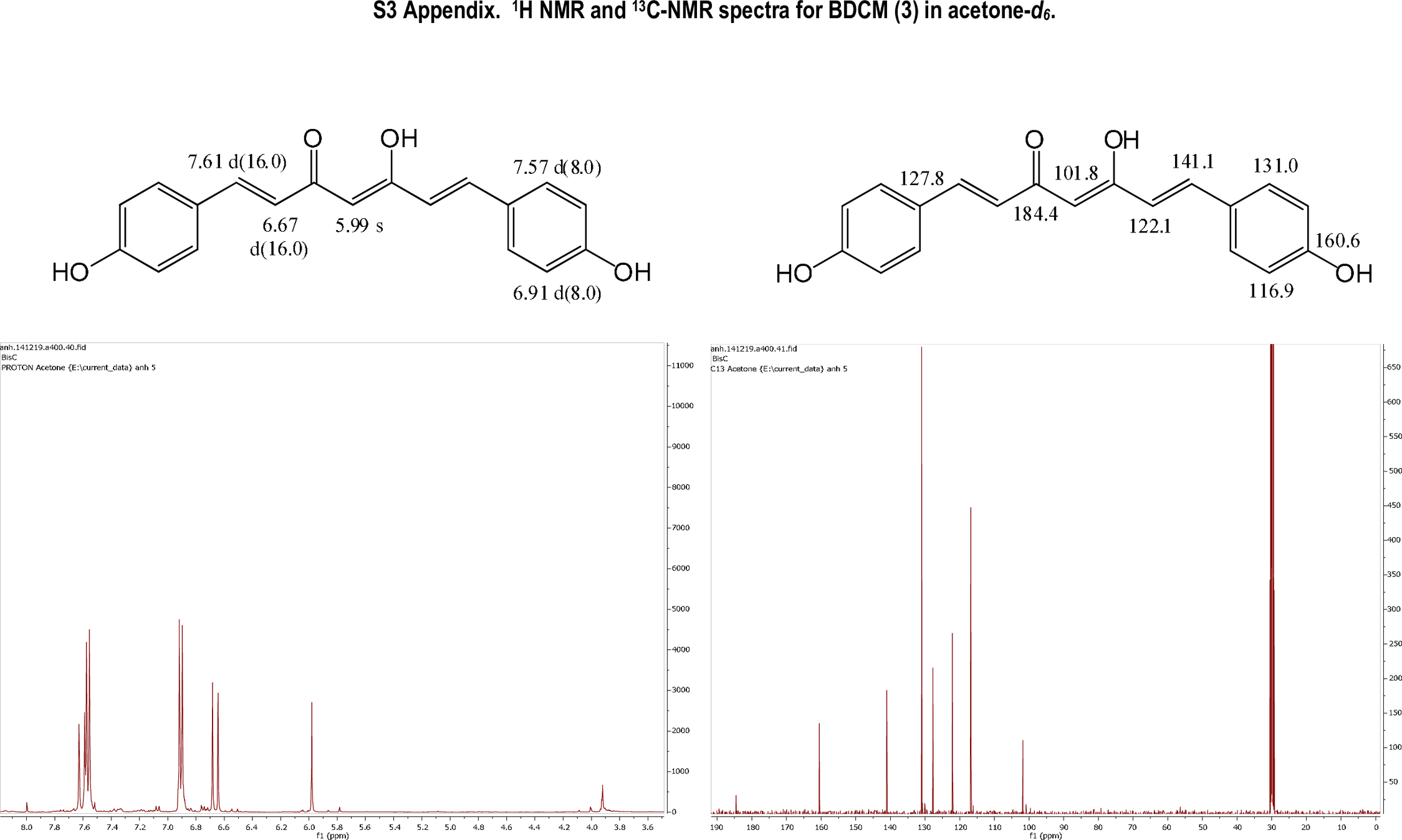

Supplement: S3 Appendix — (TIF) [file pone.0163260.s003.tif]

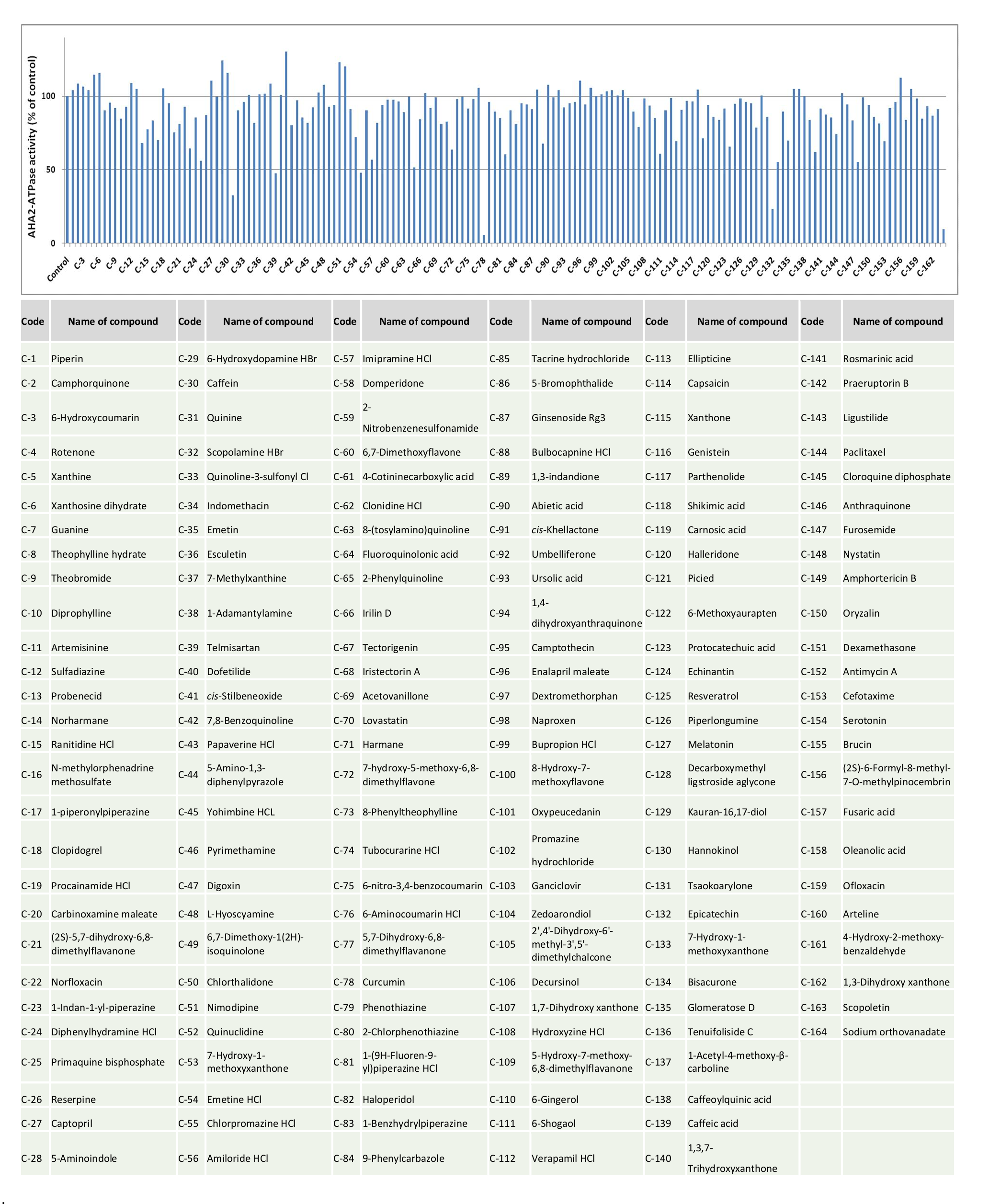

Supplement: S1 Fig — ATPase activity measured in the presence of 100 μM of the indicated compounds. The effect is presented relative to an untreated sample. A sample containing the well-described inhibitor of P-type ATPases, vanadate, is included as control. Below the diagram all tested compounds are listed. (TIFF) [file pone.0163260.s004.tiff]

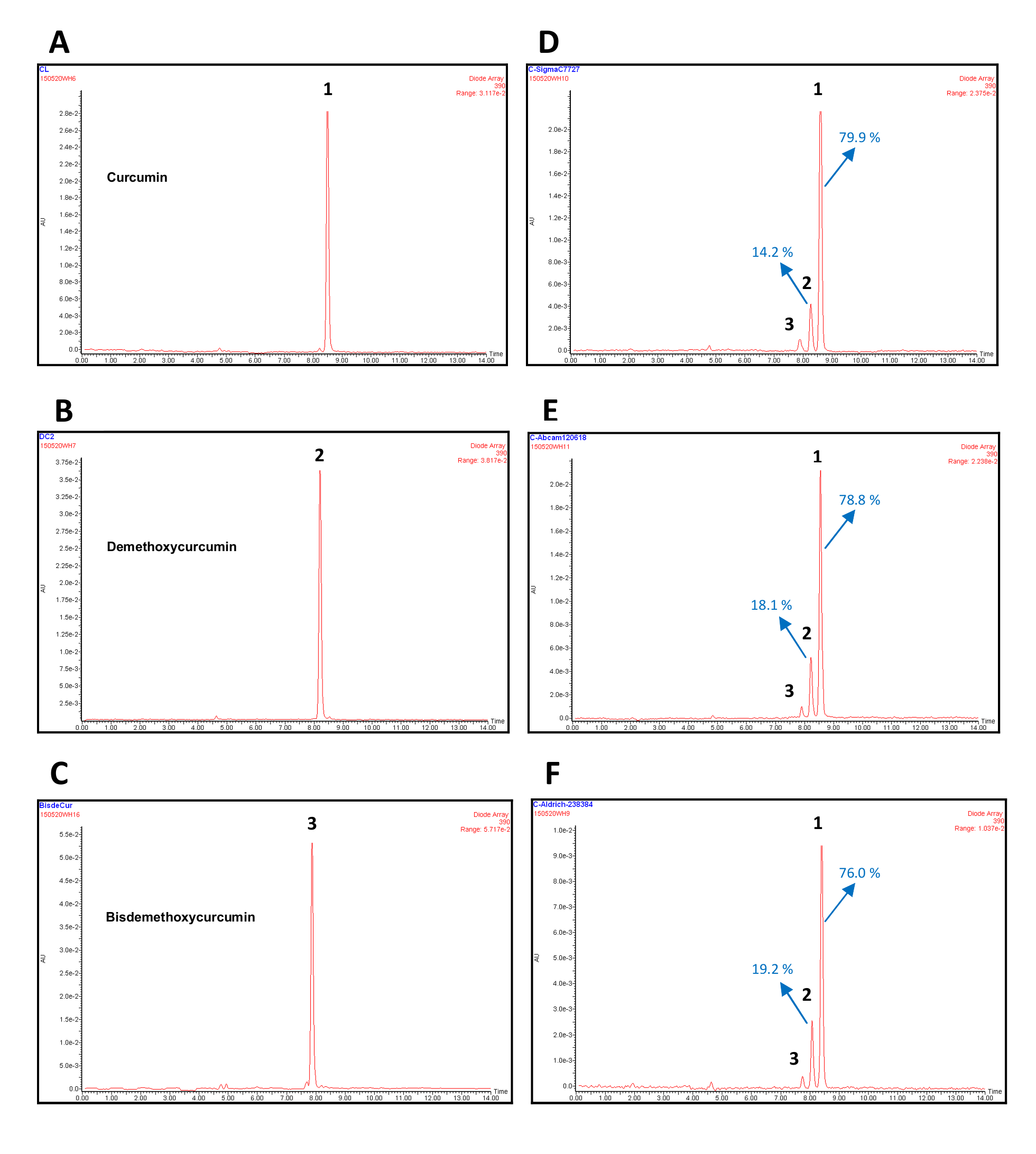

Supplement: S2 Fig — Analytical HPLC profiles of compounds 1–3 (A-C), and curcumins Sigma #C7727 (D), Abcam #120618 (E) and Aldrich #238384 (F) were performed using a Waters system: DAD detector 390 nm, column RP C18 (150 x 4.6 mm), flow rate 0.8 mL/min, injection volumn 5 μL of 0.1 mg/min sample solution, solvent gradient 44.9% MeCN, 55% H2O and 0.1% formic acid to 98.9% MeCN, 1% H2O and 0.1% formic acid over 15 min. (TIFF) [file pone.0163260.s005.tiff]
